# Comparison of Human Neonatal and Adult Blood Leukocyte Subset Composition Phenotypes

**DOI:** 10.1371/journal.pone.0162242

**Published:** 2016-09-09

**Authors:** Savit B. Prabhu, Deepak K. Rathore, Deepa Nair, Anita Chaudhary, Saimah Raza, Parna Kanodia, Shailaja Sopory, Anna George, Satyajit Rath, Vineeta Bal, Reva Tripathi, Siddharth Ramji, Aruna Batra, Kailash C. Aggarwal, Harish K. Chellani, Sugandha Arya, Nidhi Agarwal, Umesh Mehta, Uma Chandra Mouli Natchu, Nitya Wadhwa, Shinjini Bhatnagar

**Affiliations:** 1 Pediatric Biology Center, Translational Health Science and Technology Institute, Faridabad, Haryana, India; 2 National Institute of Immunology, New Delhi, India; 3 Department of Obstetrics & Gynecology, Maulana Azad Medical College, New Delhi, India; 4 Department of Neonatology, Maulana Azad Medical College, New Delhi, India; 5 Department of Obstetrics & Gynecology, Vardhman Mahavir Medical College and Safdarjung Hospital, New Delhi, India; 6 Department of Pediatrics, Vardhman Mahavir Medical College and Safdarjung Hospital, New Delhi, India; 7 Department of Obstetrics and Gynecology, Gurgaon Civil Hospital, Gurgaon, India; 8 Department of Pediatrics, Gurgaon Civil Hospital, Gurgaon, India; Massachusetts General Hospital, UNITED STATES

## Abstract

The human peripheral leukocyte subset composition depends on genotype variation and pre-natal and post-natal environmental influence diversity. We quantified this composition in adults and neonates, and compared the median values and dispersal ranges of various subsets in them. We confirmed higher frequencies of monocytes and regulatory T cells (Tregs), similar frequencies of neutrophils, and lower frequencies of CD8 T cells, NKT cells, B1 B cells and gamma-delta T cells in neonatal umbilical cord blood. Unlike previous reports, we found higher frequencies of eosinophils and B cells, higher CD4:CD8 ratios, lower frequencies of T cells and iNKT cells, and similar frequencies of CD4 T cells and NK cells in neonates. We characterized monocyte subsets and dendritic cell (DC) subsets in far greater detail than previously reported, using recently described surface markers and gating strategies and observed that neonates had lower frequencies of patrolling monocytes and lower myeloid dendritic cell (mDC):plasmacytoid DC (pDC) ratios. Our data contribute to South Asian reference values for these parameters. We found that dispersal ranges differ between different leukocyte subsets, suggesting differential determination of variation. Further, some subsets were more dispersed in adults than in neonates suggesting influences of postnatal sources of variation, while some show the opposite pattern suggesting influences of developmental process variation. Together, these data and analyses provide interesting biological possibilities for future exploration.

## Introduction

Human populations show considerable inter-individual diversity in immune phenotype and function[1,2]. Such population-level-variation in immune phenotypes plausibly maintains plasticity in response to varying environmental pathogenic challenges and may help in adaptation to newly emerging pathogenic threats[3]. Diversity in the phenotypes of leukocyte proportions and concentrations in blood is thought to have variable contributions from genetic[4,5], environmental[6–8] and epigenetic[9–11] factors. There is evidence that socio-geographic differences have consequence in immune phenotype and function[12–15]. Such differences across countries could possibly be attributed to differences in genetic structure, microbial-antigenic loads or nutritional status[16] of the population. Epidemiological studies show that, even in an ethnically and geographically homogenous population, immune parameters show variation from individual to individual. At an individual level, responsiveness to environmental fluctuations is essential for immunity; hence recent environmental exposures as well as lifetime cumulative exposure to antigenic stressors may well affect steady state blood leukocyte numbers and concentrations. Genetic heritable factors also modify individual responses in physiology and disease [1,2,17]. Genetic studies using twins and genome-wide association studies have identified some of the genetic loci associated with various immune phenotypes, albeit with some conflicting results [4,6,18].

These modulating determinants can potentially affect any stage of immune system development and function. The immune system has a variety of lineages and cell types with different differentiation histories and rates of maturation. Hence it is possible that the relative contributions of genetic, epigenetic and environmental modulators of variation may vary from lineage to lineage. It is also possible that different lineages/sub-lineages have differing inherent sensitivities for environmental fluctuations and nutritional states. The extent and determinants of such diversity for immune cell lineages is thus a matter of considerable interest. Interestingly, at birth a normal neonate would be expected to have relatively little environmental exposure. Hence it is plausible that variation in specific leukocyte lineages present in neonatal umbilical cord blood may be more rooted in differences in differentiation histories of the lineage during embryogenesis, while environmental factors would be expected to contribute strongly to variability observed in adults.

To explore these issues further, we have performed extensive flow cytometric phenotyping of adult peripheral blood leukocytes as well as neonatal umbilical cord blood leukocytes. While reference values for the major immune cell categories that are necessary for clinical purposes have been generated for both adults and neonates[19,20], such data are not easily available, especially in non-Western settings, for many recently defined immune cell subsets. Also, these data allow us to identify new interesting differences in certain specific leukocyte subsets between adults and neonates. Our data further show that the extent of dispersal between individuals varies widely for different blood leukocyte subsets. Further, to explore the possible basis of quantitative diversity in leukocyte subsets, we compare the dispersal of leukocyte subsets between adults and neonates. Our data provide interesting differences between adults and neonates with regard to specific blood leukocyte subsets, and identify specific leukocyte subsets that are more broadly dispersed in either adults or neonates, providing potential for further work to elucidate the biological bases of population-level variation in immune phenotypes.

## Methods

### Study design and sample processing protocols

This was an analytical cross-sectional case-control study. The outcome of interest was characterization of frequencies and concentrations of various leukocyte cell lineages and subsets. For sample size calculation, on the basis of preliminary data, we used the ratio of CD4 to CD8 T cell frequencies in adult blood (AB) and full-term neonatal umbilical cord blood (CB) (1.60 +/- 0.78 and 2.14 +/- 1.14 respectively) for detection of differences with 90% power at p = 0.05 level of significance (two-sided). The sample size required was sixty-nine AB and CB samples.

Adult volunteers were healthy adults between the ages of 18 to 55 years, with no illness at the time of blood collection. Volunteers with any documented chronic infectious or non-infectious diseases, medication intake, immunization within the last 4 weeks, hospitalization in the last 3 months, blood transfusion in the last 6 months and female volunteers who were pregnant were excluded. Ten ml AB was collected from an antecubital vein into heparinized tubes and transported immediately to the laboratory.

CB was collected from full-term neonates from healthy mothers delivered by normal vaginal delivery. Neonates who had any maternal antenatal risk factors were excluded. Twenty-five ml CB was collected immediately after delivery of the placenta from the umbilical cord vein, by venipuncture at the cut end of the cord attached to the placenta. Blood for flow cytometry was dispensed into heparinized tubes and transported immediately to the laboratory.

All samples were processed and stained within 3 h of delivery and analyzed flow cytometrically within 12 h after staining. Total leukocyte counts were estimated using standard clinical hematology laboratory procedures.

The study was reviewed and approved by the institutional human ethics committees of participating institutions [(1) Institutional Ethics Committee (Human Research), Translational Health Science and Technology Institute—Ethics/THS/1.8.1(4) dated 08-10-2012, (2) Ethics committee, Moulana Azad Medical College—F.2/IEC/MAMC/11/No 63 dated 01-03-2012, (3) Ethics committee, V.M. Medical College and Safdarjang hospital- No.47-11-EC(32/51) dated 13-01-2012 and (4) Institutional Ethics Committee, Gurgaon general hospital- Ethics/GGH/2012/1.4 dated 19-10-2012)]. The methodologies used in the study were in accordance with approved guidelines. All experimental protocols used in this study were approved by Institutional Ethics Committee (Human Research) of Translational Health Science and Technology Institute.

Informed written consent was taken from all adult volunteers before phlebotomy. For cord blood samples, informed written consent was taken from mother during the ante-natal period itself. Eligible and willing participants (adult volunteers and mothers of neonates) were given a detailed written and verbal information about the study in vernacular language and informed written consent was taken (by thumb print in the case of illiterates) in the presence of a witness.

The neonatal samples reported in this study are a subset of the neonatal samples assayed and reported previously [21]. Those full-term normal-weight neonatal samples that were obtained over the same time period and analyzed in tandem with the adult samples were included here.

### Flow cytometry

Ammonium chloride was used to lyse erythrocytes from whole blood, as described previously [22]. Leukocytes were washed and stained using five different cocktails for flow cytometry. The following fluorochrome-labeled monoclonal antibodies were used: CD45 (2D1), CD16 (3G8), CD14 (M5E2), CD11c (B-ly6), CD56 (NCAM16.2), CD25 (M-A251), CD3 (OKT-3), CD4 (RPA-T4), CD8 (RPA-T8), TCRgamma/delta (g/d) (B1), CD45RA (HI100), CD62L (DREG-56), CD19 (HIB19), CD20 (2H7), CD27 (M-T271), lineage (LIN: CD3, CD14, CD16, CD19, CD20, CD56), HLA-DR (L243), CD123 (7G3) (all from BD Biosciences San Jose, CA), CD66b (G10F5), CD163 (GHI/61), TCR Va24-Ja18 (6B11), CD43 (10G7), CD10 (HI10a) (all from BioLegend Inc. San Diego, CA). Each staining well contained 3 million cells, and staining was done on ice for 30 minutes, followed by washing in PBS. Cells were fixed in 2% paraformaldehyde and analyzed flow cytometrically (FACSCanto II or FACSAria III, BD Biosciences, San Jose, CA), using FlowJo (TreeStar, Ashland, OR).

### Statistical analysis

Comparison of AB and CB for the various leukocyte subsets was performed using the Mann-Whitney test with no assumption of normality, and a p value of <0.05 was considered significant. Comparison of relative variation was carried out using the Brown-Forsythe test after normalizing each dataset against its median, and a p value of <0.05 was considered significant.

## Results

**Fig 1:**
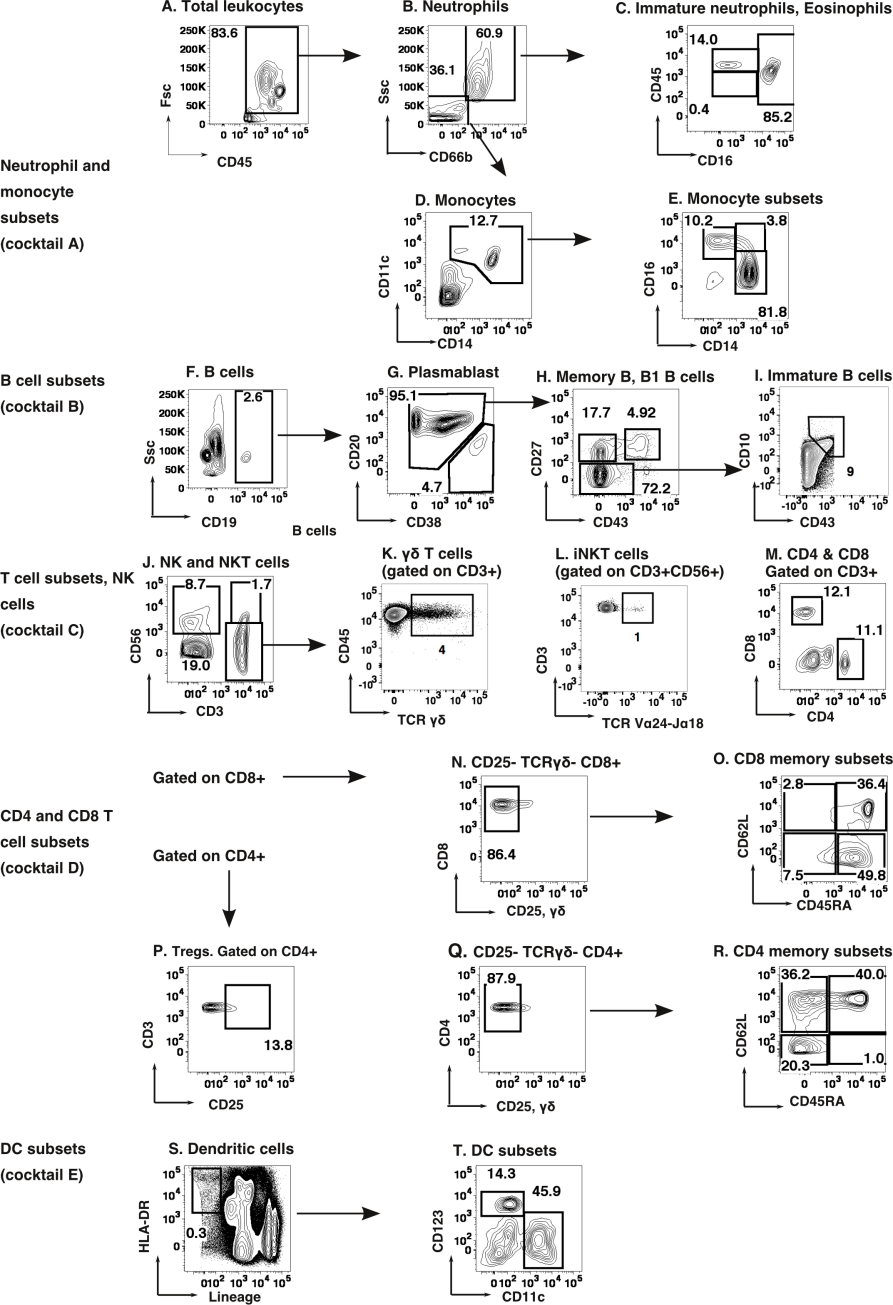
Flow cytometric gating strategies used for leukocyte subset identification using marker molecules (indicated in Table 3). On CD45+ total leukocytes (A), CD66b was used to distinguish Monocytes (CD66b –ve) (Fig 1D) and CD66b +ve population which consists of Neutrophils (CD66b+ CD16+), immature Neutrophils (CD66b+ CD16- CD45 dull) and Eosinophils (CD66b+ CD16- CD45 bright) (Fig 1B and 1C). Monocytes were classified into Classical (CD14+ CD16-), Inflammatory (CD14+ CD16+) and patrolling (CD14 dull CD16+)(Fig 1D and 1E). B cells were gated as CD19+ cells (Fig 1F). Plasmablasts (CD19+ CD20 dull CD38+) were excluded (Fig1G). Remaining B cells were further gated as memory B (CD19+CD27+CD43-), naïve B (CD19+CD27-) and B1 B (CD19+CD27+CD43+) cells (Fig 1H). Immature B cells (Fig 1I) were gated as CD10+CD43+ on naïve B cells (Fig 1H). In the NK and T cell cocktail CD3-CD56+ cells are NK cells whereas CD3+CD56+ cells are NKT cells (Fig 1J). CD3+CD56- cells (Fig 1J) were further analysed for **γδ** T cells (Fig 1K), CD4 and CD8 T cells (Fig 1M). iNKT cells are gated on NKT cell population (Fig 1L). CD25-TCR**γδ-** CD8+ cells were further subdivided into memory subsets (Fig 1O). While Tregs were identified as CD25+ cells of CD4 population (Fig 1P), CD25-TCR **γδ**- CD4 cells (Fig 1Q) were further subdivided into memory subsets (Fig 1R). DCs were identified as lineage-HLA-DR+ cells(Fig 1S) and further subsets were characterized as shown in Fig 1T. For cell populations that do not show a distinct contour, FMO controls are illustrated in S1 Fig.

The demographic characteristics of the neonates and adult volunteers are presented (Table 1 and Table 2). The gating strategies used for flow cytometric identification of the leukocyte subsets (Fig 1 and Table 3) were adopted following previous reports [21]. Twenty-nine subsets, at various levels of classification, of total CD45-expressing leukocytes were thus enumerated.

**Table 1:** Characteristics of participant population- cord blood.

Characteristics ^*a*^		Cord Blood (n = 76)
Parental		
Mother’s age, (years)		24.1 (2.8)
Father’s age, (years)		27.8 (3.2)
Mother’s intrapartum weight, kg (n = 47)		61.6 (7.5)
Mother’s height, cm (n = 60)		155.2 (4.6)
Neonatal		
Female	n(%)	35 (46.1)
Gestational age at birth, (weeks)		39 (1.00)
APGAR score (1 minute)		8.6 (0.49)
APGAR score (5 minute)		9.0 (0.26)
Birth weight, kg		3.0 (0.3)
Length, cm		50.8 (2.2)
Head circumference, cm		34.1 (1.0)

^*a*^ All values are Mean (SD) except where specified

**Table 2:** Characteristics of Adult volunteers.

Characteristics ^*a*^		Adult Blood (n = 71)
Age, (years)		30.0 (6.2)
Female	n (%)	22 (31.0)

^*a*^ All values are Mean (SD) except where specified

**Table 3:** Flow cytometric marker molecule combinations used to identify various leukocyte subsets.

Cell subset	Phenotypic markers
Total leukocytes	CD45+
Neutrophils	CD66b+ CD16+
Immature neutrophils	CD66b+ CD16—CD45 dull
Eosinophils	CD66b+ CD16- CD45 bright
B cells (excluding plasmablasts)	CD19+ CD20 bright CD38 dull
Plasmablasts	CD19+ CD20 dull CD38+
Memory B cells	CD19+ CD20 bright CD38 dull CD27+
B1 B cells	CD19+ CD20 bright CD38 dull CD27+ CD43+
Naïve B cells	CD19+ CD20 bright CD38 dull CD27-
Immature naïve B cells	CD19+ CD20 bright CD38 dull CD27- CD10+ CD43+
Total monocytes	CD66b- CD14+ CD11c+
Classical monocytes	CD66b- CD14+ CD11c+ CD16-
Patrolling monocytes	CD66b- CD14 intermediate CD11c+ CD16+
Inflammatory monocytes	CD66b- CD14+ CD11c+ CD16+
Total T cells	CD3+
Gamma-delta T cells	CD3+ γδ TCR+
NK cells	CD56+
NKT cells	CD3+ CD56+
iNKT cells	CD3+ CD56+ TCR Va24-Ja18
CD4 T cells	CD3+ CD4+
CD8 T cells	CD3+ CD8+
Tregs	CD3+ CD4+ CD25+
Central memory CD4 T cells	CD3+ CD4+ CD45RA- CD62L+
Effector memory CD4 T cells	CD3+ CD4+ CD45RA- CD62L-
CD4 EMRA	CD3+ CD4+ CD45RA+ CD62L-
Naïve CD4 T cells	CD3+ CD4+ CD45RA+ CD62L+
Central memory CD8 T cells	CD3+ CD8+ CD45RA- CD62L+
Effector memory CD8 T cells	CD3+ CD8+ CD45RA- CD62L-
CD8 EMRA	CD3+ CD8+ CD45RA+ CD62L-
Naïve CD8 T cells	CD3+ CD8+ CD45RA+ CD62L+
Dendritic cells (DC)	Lineage- HLA-DR bright
Myeloid DC	Lineage- HLA-DR bright CD11c+ CD123-
Plasmacytoid DC	Lineage- HLA-DR bright CD11c- CD123+

### Comparison of adult and neonatal leukocyte phenotypes

**Fig 2:**
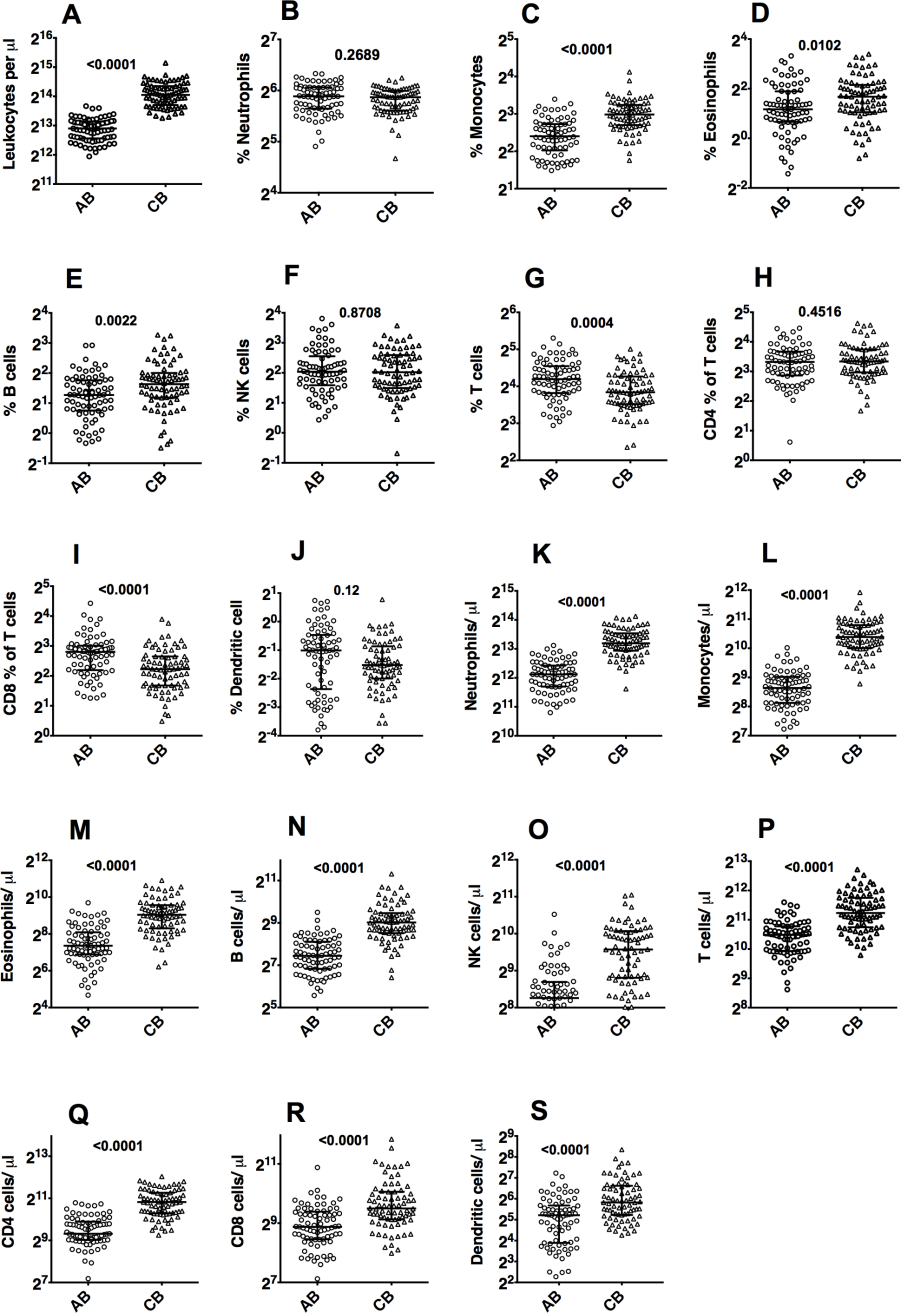
Frequencies and concentrations (cells/μl of blood) of major leukocyte subsets in AB versus CB. Numbers in the plots indicate p-values.

The total leukocyte concentration (TLC) was higher in CB than in AB (Fig 2A) as expected [19]. CB showed higher frequencies of monocytes (Fig 2C), eosinophils (Fig 2D) and B cells (Fig 2E) and lower frequencies of total T cells (Fig 2G) and CD8 T cells (Fig 2I) in comparison to AB. The frequencies of neutrophils (Fig 2B), NK cells (Fig 2F), CD4 T cells (Fig 2H) and dendritic cells (Fig 2J) were similar. Since the concentrations of all these major subsets were higher in CB (Fig 2K to 2S) as would be expected from the TLC differences, only the cell frequencies for further cellular subsets are further described here. However, the calculated cell concentrations per μL blood for all leukocyte subsets for AB and CB are also provided for reference (Table 4 and Table 5).

**Table 4:** Frequencies of AB and CB subsets.

Cell subset				Adult			Cord		
				25th percentile	median	75th percentile	25th percentile	median	75th percentile
**Monocytes, neutrophils, eosinophils**									
	Monocytes (% total leukocytes)			4.21	5.28	6.6	6.56	7.91	9.32
		Classical monocyte (% Monocytes)		70.7	74.7	79.1	81.53	85.55	89.6
		Inflammatory monocyte (% Monocytes)		2.46	3.41	4.79	2.56	3.22	4.5
		Patrolling monocyte(% Monocytes)		9.17	12	14.6	3.29	4.81	7.32
	Neutrophils (% total leukocytes)			50.24	59.14	66.65	48.85	58.42	63.7
		Immature neutrophil (% Neutrophils)		0.35	0.61	1.45	3.02	4.94	8.72
	Eosinophils (% total leukocytes)			1.59	2.25	3.69	2.08	3.2	4.42
**T cell subsets**									
	T cells (% total leukocytes)			14.2	18.3	22.9	11.55	14.3	18.88
		CD4 (% T cells)		7.4	10	12.7	7.83	10.1	13.33
			CD4 EMRA(%CD4)	0.48	0.89	1.82	nil		
			Memory CD4 (%CD4)	49.6	61.8	72.3	nil		
			Tregs (%CD4)	5.11	7.39	9.7	8.29	10.95	13.13
		CD8 (% T cells)		4.62	6.97	7.9	3.2	4.68	6.26
			CD8 EMRA (%CD8)	6	13.1	22.5	nil		
			Memory CD8 (%CD8)	51.8	65.1	74.6	nil		
		γδTcell (% T cells)		6.14	9.44	12.5	2.43	3.22	4.28
		NKT cells (% T cells)		0.027	0.054	0.089	0.007	0.014	0.026
		iNKT cells (% T cells)		0.003	0.005	0.012	0.0007	0.001	0.002
	NK cells (% total leukocytes)			3.06	4.1	5.6475	2.87	4.06	5.98
**B cell subsets**			** **						
	B cells (% total leukocytes)			1.69	2.41	3.38	2.27	3.1	4
		B1 B cells (% B cells)		2.88	3.51	4.86	1.28	1.72	2.51
		Immature B cells (% B cells)		1.115	2.1	3.66	6.28	9.24	13.7
		Memory B cells (% B cells)		15.3	22.5	27.75	nil		
		Plasmablasts (% B cells)		1.63	2.6	4.53	nil		
**Dendritic cell subsets**			** **						
	Dendritic cells (% total leukocytes)			0.19	0.49	0.72	0.25	0.35	0.55
	mDC (% total leukocytes)			0.07	0.14	0.23	0.04	0.06	0.09
	pDC (% total leukocytes)			0.03	0.05	0.09	0.03	0.06	0.09

**Reference values for cell frequencies of immune cell subsets in adult blood (AB) and cord blood (CB).** Frequencies of monocytes, neutrophils, eosinophils, T cells, B cells, NK cells, dendritic cells, mDC and pDC are expressed as frequency of total leukocytes (CD45+ cells). The three monocyte subsets (Classical, Inflammatory and Patrolling) are expressed as frequency of monocyte population. Immature neutrophils are expressed as frequency of total neutrophils. CD4 T cell, CD8 T cell, NKT cell, iNKT cell and gamma-delta T cell frequencies are expressed as percentage of total T cells. CD4 and CD8 memory subsets are described as frequency of total CD4 and CD8 T cells respectively. Tregs are calculated as frequency of CD4 T cells. Memory B cells, immature B cells, plasmablasts and B1 B cells are calculated as percentage of total B cells

**Table 5:** absolute counts of cell subsets.

	Adult			Cord		
(Expressed as ‘per μl’ of blood)	25th percentile	median	75th percentile	25th percentile	median	75th percentile
Total Leukocyte count	6090	7650	8950	13575	16950	20825
**Monocytes, neutrophils, eosinophils**						
Monocytes	279.08	397.85	515.94	1032.51	1338.42	1773.72
Classical monocyte	209.18	302.98	385.05	884.98	1160.95	1467.16
Inflammatory monocyte	7.23	14.16	21.43	26.96	45.4	66.03
Patrolling monocyte	28.8	43.33	64.47	38.25	61.25	117.04
Neutrophil	3325.51	4453.84	5426.95	7910.52	9353.05	11867.3
Immature neutrophil	15.76	29.6	65.96	260.68	472.14	908.55
Eosinophil	118.61	164.03	265.64	329.3	526.1	746.23
**T cell subsets**						
Tcells	975	1432.8	1744.2	1725.67	2402.38	3406.33
CD4	520.68	637.57	924.15	1260.28	1808.79	2440.92
CD4 EMRA	2.81	6.62	13.34	nil		
Memory CD4	270.48	380.99	492.83	nil		
Tregs	32.52	44.81	77.17	125.23	173.19	243.2
CD8	353.5	469.03	661.5	558.54	721.8	1064.62
CD8 EMRA	24.52	58.55	125.09	nil		
Memory CD8	191.25	273.18	369.96	nil		
γδTcell	78.94	130.36	195.75	56.6	78.91	98.43
NKT cells	0.34	0.79	1.21	0.17	0.31	0.71
iNKT cells	0.03	0.06	0.19	0.02	0.03	0.05
**NK cells**	218.03	306.25	410.81	450	760.79	1059.94
**B cell subsets**						
B cells	115.36	173.99	267.22	366.86	518.94	697.17
B1 B cells	3.87	6.11	8.71	6.27	9.11	15.26
Immature B cells	0.98	2.58	4.04	25.4	34.15	66.92
Memory Bcells	19.89	31.39	57.21	nil		
Plasmablasts	2.6	4.6	8.1	nil		
**Dendritic cell subsets**						
Dendritic cells	14.82	37.12	51.2	37.38	56.54	98.43
mDC	6.02	10.24	17.02	6.23	9.49	14.52
pDC	2.41	3.55	6.09	5.55	8.14	14.18

**Reference values for concentrations of immune cell subsets in adult blood (AB) and cord blood (CB).** All counts are per μl of blood.

**Fig 3:**
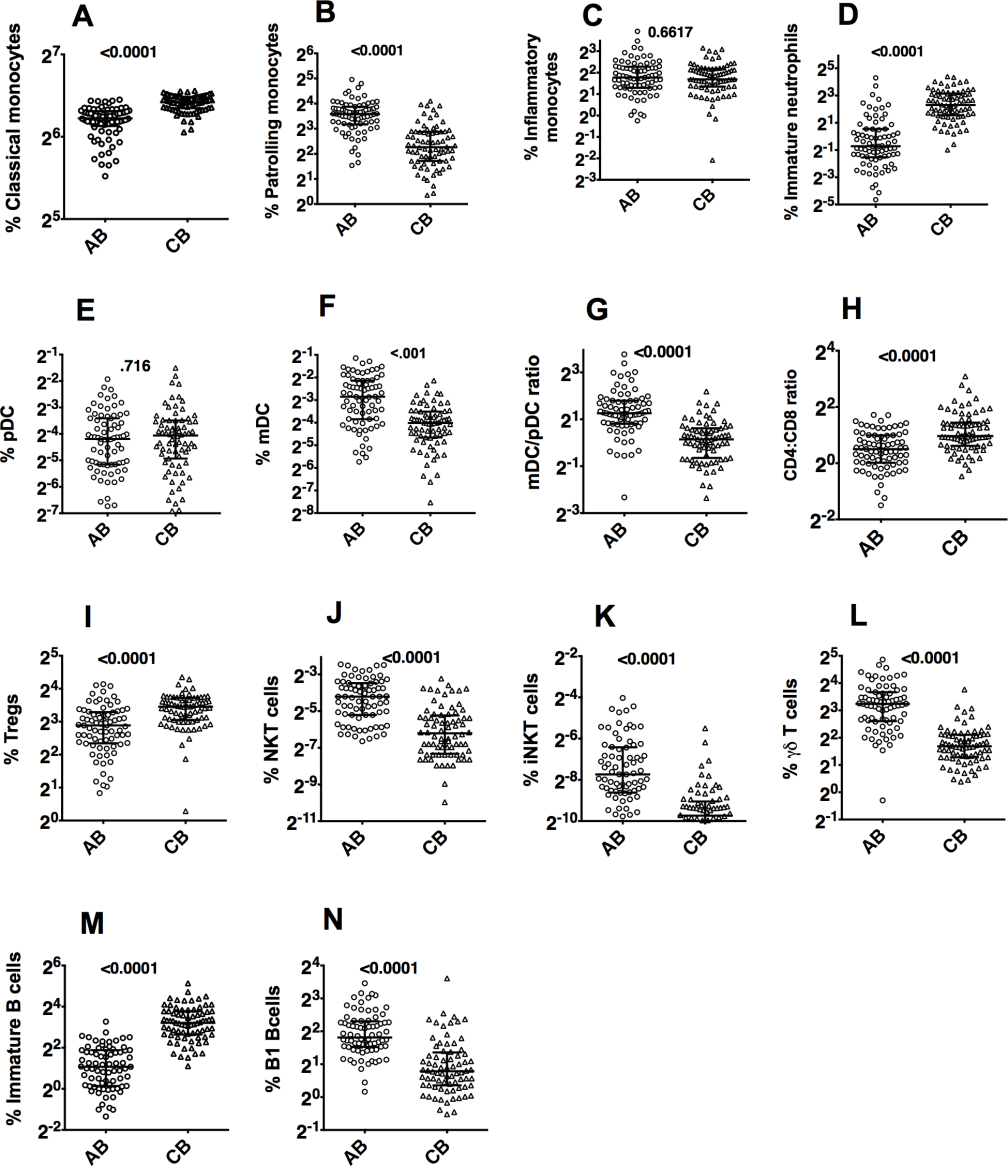
Frequencies of leukocyte sub-lineages in AB versus CB. Values in the plots indicate p-values.

Among the monocyte cell subsets, CB showed higher frequencies of classical monocytes and lower frequencies of patrolling monocytes compared to AB (Fig 3A and 3B). The frequencies of inflammatory monocytes were similar (Fig 3C). In the dendritic cell lineage (Fig 3E to 3G), CB showed lower ratios of myeloid dendritic cells (mDCs) to plasmacytoid dendritic cells (pDCs) compared to AB (Fig 3G). The frequencies of immature neutrophils were significantly higher in CB compared to AB (Fig 3D).

In the T cell lineage, CB showed higher CD4/CD8 ratios, higher frequencies of putative regulatory T cells (Tregs), lower frequencies of gamma-delta T cells, NKT cells and iNKT cells (Fig 3H to 3L). As expected, there were no discrete populations of memory T or B cells or plasmablasts detectable in CB. We have used gating using CD45RA and CD62L to differentiate naïve, central memory, effector memory and EMRA T cells (Table 2). Among B cell subsets, B1 B cell frequencies were lower in CB (Fig 3N) and immature naïve B cell frequencies were higher in CB compared to AB (Fig 3M).

A comparison between males and females of cord and adult samples is given in S1 Table. However, the sample size is not big enough to permit definitive interpretations.

### Population-level variation in the peripheral leukocyte phenotype between neonatal and adult populations and between various leukocyte lineages

**Fig 4:**
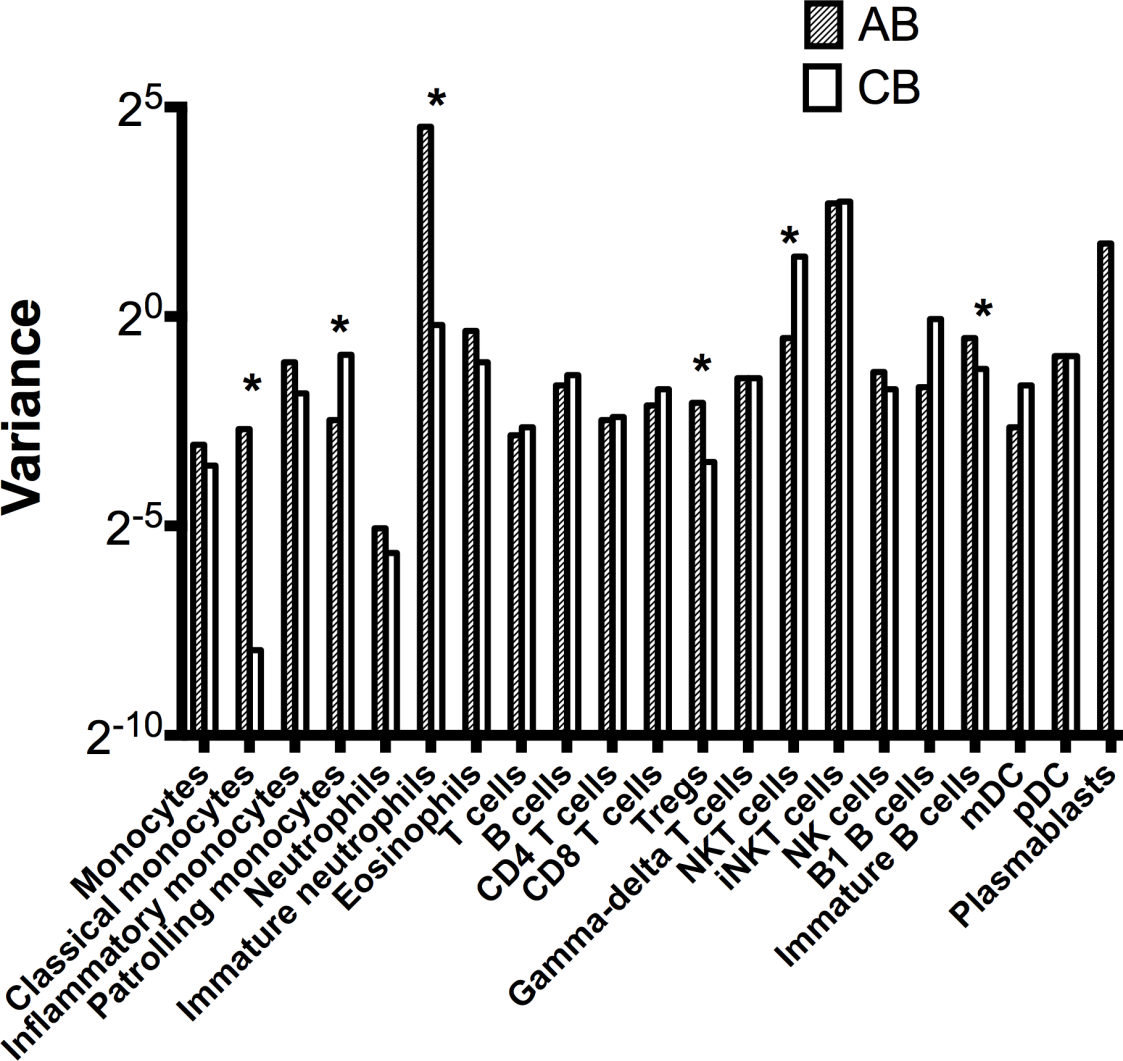
Variances in AB and CB in various leukocyte lineages. Values on y-axis are median-corrected variances. Asterices mark p<0.05 (Brown-Forsythe test).

As a measure of the dispersal of the values for each leukocytic lineage, we examined relative variances for all lineage frequencies in AB and CB, using median-normalized variances so as to account for differences in median values. The dispersal of values for most leukocyte subsets was strikingly similar between AB and CB (Table 6). However, for some subsets (Tregs, immature neutrophils, immature B cells and classical monocytes), the variation was higher in AB than in CB (Fig 4), while it was higher in CB than in AB for NKT cells and patrolling monocytes (Fig 4).

**Table 6:** Median-corrected variances and p-values (Brown-Forsythe test) of leukocyte subsets in AB and CB.

	Median-corrected variance		
Frequencies of:	AB	CB	p-value (Brown-Forsythe test)
Monocytes	0.12	0.085	0.051
Classical monocytes	0.155	0.004	0.0008
Inflammatory monocytes	0.47	0.28	0.33
Patrolling monocytes	0.18	0.53	0.0034
Neutrophils	0.03	0.02	0.136
Immature neutrophils	23.1	0.87	0.009
Eosinophils	0.79	0.47	0.27
T cells	0.14	0.16	0.77
B cells	0.32	0.38	0.72
CD4 T cells	0.18	0.19	0.67
CD8 T cells	0.23	0.3	0.22
Tregs	0.24	0.09	0.0006
Gamma-delta T cells	0.36	0.36	0.4
NKT cells	0.7	2.7	0.013
iNKT cells	6.5	6.7	0.28
NK cells	0.4	0.3	0.99
B1 B cells	0.31	0.96	0.15
Immature B cells	0.7	0.42	0.048
mDC	0.16	0.32	0.06
pDC	0.52	0.52	0.9
Plasmablasts	3.35		

Interestingly, the degree of population-level dispersal was also different between different leukocyte lineages. For example, the frequencies of plasmablasts or of immature neutrophils, which may reflect transient recent microbial exposure, showed far greater variation in the adult population than classical monocytes (Fig 4).

## Discussion

We have characterized human adult and neonatal cord blood leukocyte phenotype in detail in terms of a number of cellular sub-lineages. Our data on immune cell frequencies and cell counts will contribute to the development of reference standards for the sampled ethno-geographic population in this study. One of the limitations of this study is that early pediatric age groups are not studied. This is important since the major changes in immune phenotypes occur during this time. The comparison of leukocyte subsets between AB and CB reveals some interesting findings. Some of the differences observed here are consistent with previous reports, such as higher TLC values [19], higher monocytes [19,20], lower CD8 T cells [20,23], lower NKT cells [20,23–25], lower gamma-delta T cells [20,24], higher Tregs [26], lower B1 B cells [27] and similar neutrophils [19] in CB. However, it must be noted that for identifying Treg cells we have not used FoxP3 as a marker. While in CB population it is not a major problem, in AB identification of Treg population as CD4+CD25+ is likely to score recently activated CD4 T cells in addition to Treg cells thus, somewhat spuriously increasing the effective frequency of Treg cells. The higher eosinophil, lower iNKT cell and similar CD4 T cell frequencies and higher CD4/CD8 ratios that we find in CB differ from earlier reports [19,20,25,28]. With regard to the differences that are observed in T cells (lower in CB), B cells (higher in CB) and NK cells (similar in CB and AB), our study either corroborates [20,25,29] or differs from [20] the existing literature. However, we present these data not as a comparison between populations but as reference values for the population we studied.

Although monocyte subsets [30,31] and DC subsets [32,33] have been characterized in CB previously, they follow older terminology and classification strategies. In one previous study, monocyte subset frequencies as defined by CD14 and CD16 markers (classical monocytes-CD14++ CD16- and inflammatory monocytes-CD14+CD16+) were found to be similar in AB and CB [30] whereas another study reports inflammatory monocytes (CD14+ CD16+) to be lower in CB [31]. Both these papers do not mention patrolling monocytes. In contrast to these studies, we find predominant difference in patrolling monocytes (lower in CB) and no differences in classical or inflammatory monocytes. Similarly, for DC subsets, previous studies have used different strategies in their gating and use of surface markers to characterize DC subsets. One of them follows older terminology and gating strategy [CD1c (for myeloid DCs) and BDCA 2 and 4 (for ‘lymphoid’ DC)] and reports reduction in ‘lymphoid’ DC and similar frequency of myeloid DCs in the CB [32]. Another study that adopts a gating strategy similar to that of ours reports lower frequency of myeloid DC and higher frequency of plasmacytoid DC in the CB [33]. Our findings thus contradict the existing studies; we find similar frequencies of plasmacytoid DCs in AB and CB and lesser frequency of myeloid DC in CB. Since most of these previous studies report data from developed economies, the differences we find are likely to be promising starting points for investigating the genetic and/or environmental regulation of these traits. In addition to these we have done preliminary analysis of gender segregated differences, if any, in AB and CB cell subset frequencies. However, the sample size is not adequate for detailed analysis.

CB shows higher frequencies of two immature subsets, immature B cells and immature neutrophils, suggesting that these compartments are still maturing at birth. The absence of plasmablasts and true memory T cells suggests limited, if any, active adaptive intrauterine immune stimulation. The very low frequencies of apparently non-naïve T cells in CB are likely to be an artifact of analysis since they do not appear as clearly distinct populations.

In addition to comparing leukocyte subsets between AB and CB by measures of central tendency, we also compared the relative extent of dispersal of the data in these groups. This biological variability would be related to spatiotemporal variation in environmental exposures as well as to genetic heterogeneity. The adult and neonatal groups in our study were both from the same ethnic-geographic pool and can be expected to show similar genetic heterogeneity. However, they would differ, of course, with regard to developmental maturation states and environmental exposures. Indeed, the absence of plasmablasts and memory T and B-lymphocytes in CB was consistent with negligible microbial exposure.

When we compared data dispersal for each leukocyte subset in AB and CB, AB showed higher data dispersal in some cases, such as immature neutrophils, immature B cells, Tregs and classical monocytes. This may indicate significant influence of post-natal environmental factors on these subsets. Tregs are likely to be generated exclusively from the thymus in the neonate, whereas induced antigen-specific Tregs are also generated peripherally in response to antigenic exposure in adults, a potential additional source of heterogeneity. Neutrophil numbers vary in response to acute microbial exposure by modulation of bone marrow output [34], making it a likely explanation for greater dispersal in the AB than in the CB group. More work will be needed for an explanation for the greater relative dispersal of classical monocytes and immature B cells in AB.

Some subsets, such as NKT cells and patrolling monocytes, showed greater relative dispersal in CB. It is plausible that such leukocyte subsets show greater ‘noise’ during the developmental immature state of the neonate. The lower prominence of all these subsets in CB supports such a possibility. However, it is noteworthy that immature B cell frequencies, which could be expected to fall in the same category, did not show higher dispersal in CB.

It is remarkable that, for many leukocyte subsets, relative data dispersal was comparable between AB and CB. Heterogeneity in neonates, which would be expected to be due to genetic/epigenetic factors, appears to remain relatively unchanged in adults, in whom post-natal diversity of environmental experiences does not seem to contribute further substantial variation despite the ability of all leukocyte subsets to respond vigorously to many environmental stimuli. Thus, genetic/epigenetic factors may be major influences on leukocyte subset niche sizes.

This possibility is supported, albeit somewhat controversially, in previous work [4–6,9,10] and would be interesting to explore further.

Another notable finding in our analysis of relative data dispersal was the variation in dispersal between different leukocyte subsets. Some of these differences could be attributed to differential sensitivity of different subsets to topical environmental influences. Thus, in AB, dispersal of plasmablast frequencies is greater than that of most other leukocyte subsets. This is consistent with the transient nature of the blood plasmablast population [35]. However, variation in dispersal between different leukocyte subsets was found not only in AB, but also in CB. This may suggest evolutionary selection of niche size heterogeneity for individual leukocyte subsets.

Thus, this study contributes to the generation of reference values for leukocyte subsets in the ethno-geographic population studied, particularly in a non-Western setting. Our data also identify differences in certain specific leukocyte subsets between adults and neonates and in the extent of dispersal between individuals and between subsets. Future work will be needed to explore the potential biological insights into the basis of quantitative diversity in leukocyte subsets.

## Supporting Information

S1 Fig FMO controls for TCRγδ, TCR Vα24-Jα8, CD62L and CD43. TCRγδ, TCR Vα24-Jα8 and CD62L are gated on T cells and CD43 on naïve B cells.
(TIF)

S1 Table Comparison of frequencies and counts of adult and cord blood between males. (PDF)
