# Supplementary figures and images for: Comparison of Human Neonatal and Adult Blood Leukocyte Subset Composition Phenotypes
Source: PLoS One. 2016 Sep 9;11(9):e0162242. doi: 10.1371/journal.pone.0162242 (PMC5017693; doi:10.1371/journal.pone.0162242)

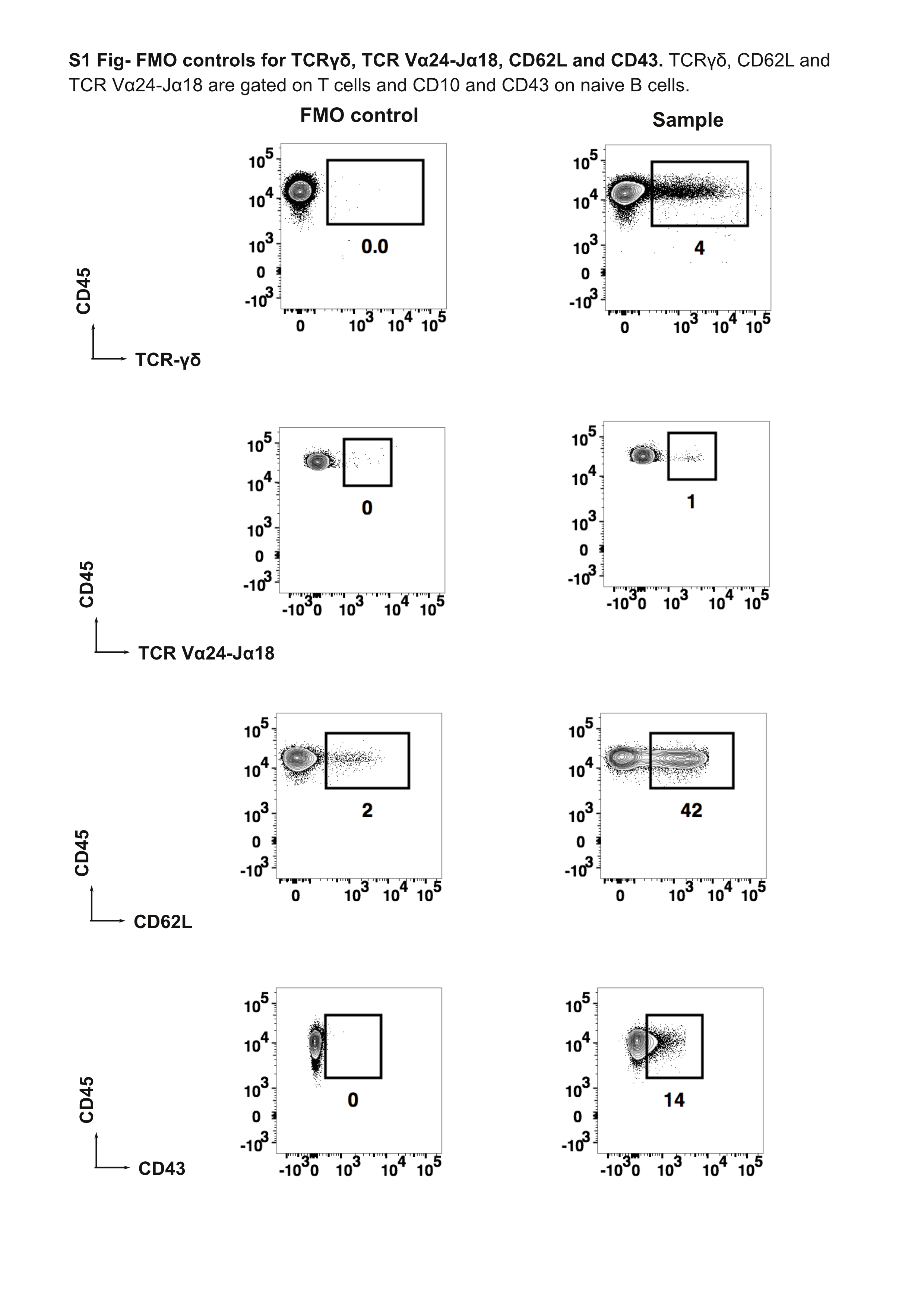

Supplement: S1 Fig — TCRγδ, TCR Vα24-Jα8 and CD62L are gated on T cells and CD43 on naïve B cells. (TIF) [file pone.0162242.s001.tif]
