# Supplementary material for: Comparison of Human Neonatal and Adult Blood Leukocyte Subset Composition Phenotypes
Source: PLoS One. 2016 Sep 9;11(9):e0162242. doi: 10.1371/journal.pone.0162242 (PMC5017693; doi:10.1371/journal.pone.0162242)
Supplement: S1 Table — (PDF) [file pone.0162242.s002.pdf]

S1 table: Comparison of frequencies and counts of adult and cord blood between males and females

**Adult blood- cell frequencies**	Male				Female				
	25% Percentile	Median	75% Percentile		25% Percentile	Median	75% Percentile		p-value
Frequency- Monocytes	4	5.6	7		3.8	5.3	6.5		0.40
Frequency- Neutrophils	48	59	66		53	62	73		0.19
Frequency- Eosinophils	1.7	2.3	4.1		0.91	1.6	2.9		0.10
Frequency- T cells	14	19	23		13	17	23		0.53
Frequency- B cells	1.6	2.4	3.4		1.8	2.8	3.5		0.51
Frequency- NK cells	3.3	4.1	6		2	3.9	5.9		0.33
Frequency- CD4 T cells	7.4	10	13		6.4	9.9	12		0.95
Frequency- CD8 T cells	4.4	6.9	7.7		5.7	7.1	9		0.82
Frequency- DC	0.21	0.48	0.84		0.13	0.31	0.59		0.10
Frequency- Classical monocytes	71	76	80		72	76	80		0.98
Frequency- Patrolling monocytes	9.4	12	15		7.4	11	14		0.39
Frequency- Inflammatory monocytes	2.4	3.4	4.6		2.5	3.5	5.1		0.37
Frequency- Immature neutrophils	0.37	0.7	2.4		0.28	0.55	1.1		0.40
Frequency- B1 B cells	2.7	3.7	5		3	3.6	4.8		0.48
Frequency- memory B cells	16	24	28		13	23	30		0.98
Frequency- immature B cells	1.1	2.3	3.8		0.98	1.3	2		0.01
Frequency- Plasmablasts	1.4	2.6	5.3		1.7	2.6	4.4		0.62

CD4:CD8 ratio	1	1.4	2		1	1.3	2		0.64
Frequency- CD4 EMRA	0.47	0.91	2		0.46	1	1.8		0.83
Frequency- CD8 EMRA	5.4	13	23		6.2	17	24		0.61
Frequency- memory CD4	56	69	74		47	55	65		0.0095
Frequency- memory CD8	54	69	78		48	59	64		0.0030
Frequency- CD4 CM	29	37	42		23	31	34		0.0038
Frequency- CD8 CM	7.1	12	17		6.1	8.4	12		0.04
Frequency- CD4 EM	19	23	29		15	19	29		0.06
Frequency- CD8 EM	23	29	41		17	26	34		0.04
Frequency- Tregs	4.7	7.5	10		5	6	9.8		0.96
Frequency- γδ Tcells	5.8	9.5	13		6.4	9.1	11		0.80
Frequency- NKT cells	0.027	0.053	0.096		0.028	0.054	0.076		0.61
Frequency- iNKT cells	0.0025	0.0048	0.011		0.0025	0.0036	0.01		0.53
Frequency- mDC	0.091	0.14	0.23		0.049	0.084	0.29		0.5
Frequency- pDC	0.031	0.058	0.094		0.023	0.057	0.1		0.9
mDC/pDC ratio	1.7	2.6	3.6		1.8	2.3	3.2		0.32

**Adult blood- cell counts (per µl of blood)**	Male				Female				
	25% Percentile	Median	75% Percentile		25% Percentile	Median	75% Percentile		p-value
TLC	5813	7325	8913		6350	8310	9200		0.14
Counts-monocytes	279	402	525		295	436	517		0.72
Counts-neutrophils	3166	4381	5338		3322	5191	6126		0.11
Counts-eosinophils	119	170	312		64	141	245		0.27

Counts-Tcells	965	1374	1682		1098	1451	1706		0.45
Counts-Bcells	112	164	240		130	231	284		0.17
Counts-NKcells	226	314	396		176	301	416		0.90
Counts-CD4	507	622	921		575	788	896		0.27
Counts-CD8	336	426	623		428	616	700		0.18
Counts-DC	16	35	57		11	29	42		0.19
Counts- Classical monocytes	204	303	407		230	346	382		0.60
Counts- Patrolling monocytes	29	44	65		27	43	59		0.71
Counts- Inflammatory monocytes	7	15	21		9	14	22		0.40
Counts- Immature neutrophils	16	30	85		14	29	49		0.63
Counts- B1 B cells	3.7	5.5	7.9		5.8	8.1	10		0.03
Counts- memory B cells	21	30	48		15	48	66		0.21
Counts- immature B cells	0.98	2.6	4.1		0.76	2.1	3.8		0.22
Counts- Plasmablasts	2.7	4.5	7.6		2.8	5.6	10		0.30
Counts- CD4EMRA	2.4	7.2	14		3.5	6.6	16		0.57
Counts- CD8EMRA	25	49	116		19	70	176		0.31
Counts- memory CD4	163	193	338		130	197	282		0.42
Counts- memory CD8	38	52	82		30	55	77		0.36
Counts- CD4 CM	100	155	210		105	135	196		0.47
Counts- CD8 CM	77	128	207		79	146	175		0.57
Counts- CD4 EM	272	381	508		238	405	473		0.47
Counts- CD8 EM	186	265	368		239	306	404		0.87
Counts- Tregs	32	44	65		35	44	83		0.55
Counts- γδ Tcells	60	134	198		96	129	187		0.72
Counts- NKT cells	0.33	0.72	1.2		0.39	0.95	1.1		0.84

Counts- iNKT cells	0.025	0.05	0.13		0.028	0.052	0.13		0.95
Counts- mDC	6.8	11	19		3.5	6.2	11		0.02
Counts- pDC	2.7	3.6	5.8		1.7	2.8	4.9		0.28

**Cord blood- cell frequencies**	Male				Female				
	25% Percentile	Median	75% Percentile		25% Percentile	Median	75% Percentile		p-value
Frequency- Monocytes	6.6	8.2	9.6		6.4	7.8	9.1		0.67
Frequency- Neutrophils	48	56	64		51	58	63		0.82
Frequency- Eosinophils	2.1	3.3	4.7		2.1	2.6	4.2		0.38
Frequency- T cells	12	15	21		11	14	17		0.16
Frequency- B cells	1.8	3.2	5.1		2.5	3	3.7		0.8
Frequency- NK cells	2.9	4.4	6.3		3.1	4	5.8		0.6
Frequency- CD4 T cells	8.2	10	15		7.6	9.9	12		0.15
Frequency- CD8 T cells	3	5	7.4		3.4	4.7	5.5		0.57
Frequency- DC	0.22	0.32	0.53		0.3	0.39	0.57		0.12
Frequency- Classical monocytes	81	85	89		81	86	90		0.84
Frequency- Patrolling monocytes	3.7	4.9	7.6		3.2	5.2	7.3		0.59
Frequency- Inflammatory monocytes	2.6	3.5	4.5		2.6	3.4	4.5		0.71
Frequency- Immature neutrophils	2.7	4.1	8.5		3.8	6.5	11		0.03
Frequency- B1 B cells	1.4	1.9	3		1.2	1.6	2.2		0.38
Frequency- memory B cells	0.67	1.6	2.8		0.64	1.4	2.3		0.66
Frequency- immature B cells	6.6	10	17		5.4	8.2	12		0.06
Frequency- Plasmablasts									
CD4:CD8 ratio	1.5	2	2.8		1.7	1.9	2.6		0.83

Frequency- CD4 EMRA	0.18	0.37	0.48		0.17	0.46	1.1		0.26
Frequency- CD8 EMRA	1.4	3.5	6		2.3	4.4	9.8		0.07
Frequency- memory CD4	3.9	6.9	11		4.8	6.9	12		0.65
Frequency- memory CD8	7.3	16	22		8.3	17	24		0.55
Frequency- CD4 CM	2.2	4	6.4		1.9	3.7	8.7		0.94
Frequency- CD8 CM	1.8	6.5	10		0.4	4.3	9		0.15
Frequency- CD4 EM	0.43	0.6	1.2		0.49	0.64	1.2		0.48
Frequency- CD8 EM	0.9	1.5	2.4		0.6	1	2.1		0.1
Frequency- Tregs	8	9.9	12		9.1	12	13		0.054
Frequency- γδ Tcells	2.4	3.3	4.4		2.5	3.1	3.9		0.89
Frequency- NKT cells	0.0063	0.013	0.023		0.0068	0.015	0.031		0.82
Frequency- iNKT cells	0.00049	0.0014	0.0024		0.00076	0.0011	0.0017		0.85
Frequency- mDC	0.039	0.062	0.088		0.04	0.062	0.079		0.92
Frequency- pDC	0.037	0.061	0.089		0.022	0.059	0.087		0.68
mDC/pDC ratio	0.62	1.1	1.5		0.76	1.1	1.7		0.6

**Cord blood- cell counts**	Male				Female				
	25% Percentile	Median	75% Percentile		25% Percentile	Median	75% Percentile		p-value
TLC	12800	16375	19813		15700	17650	22463		0.12
Counts-monocytes	1011	1273	1777		1106	1448	1855		0.35
Counts-neutrophils	6819	8756	11795		8120	10390	12514		0.17
Counts-eosinophils	293	526	746		344	538	746		0.97
Counts-Tcells	1617	2519	3698		1942	2477	3104		0.99
Counts-Bcells	307	441	792		412	531	680		0.26

Counts-NKcells	445	748	1084		467	785	1040		0.79
Counts-CD4	1216	1907	2695		1318	1655	2251		0.87
Counts-CD8	511	703	1136		634	769	957		0.43
Counts-DC	32	44	100		50	68	101		0.052
Counts- Classical monocytes	865	1039	1458		910	1228	1611		0.39
Counts- Patrolling monocytes	38	64	121		40	61	118		0.94
Counts- Inflammatory monocytes	26	45	75		31	46	69		0.87
Counts- Immature neutrophils	190	318	717		390	589	1033		0.02
Counts- B1 B cells	6.1	9.3	18		6.5	8.9	15		0.93
Counts- memory B cells	3	7.7	17		3.7	6.7	14		0.9
Counts- immature B cells	26	35	74		22	33	59		0.6
Counts- Plasmablasts									
Counts- CD4EMRA	2.7	6.4	9.9		2.6	7.2	19		0.5
Counts- CD8EMRA	15	22	47		14	34	69		0.11
Counts- memory CD4	38	57	127		29	71	133		0.86
Counts- memory CD8	13	38	100		3.2	34	97		0.51
Counts- CD4 CM	6.2	11	18		7.9	11	19		0.52
Counts- CD8 CM	5.3	11	17		4.1	8.9	19		0.48
Counts- CD4 EM	53	91	159		65	98	158		0.54
Counts- CD8 EM	43	90	137		60	113	207		0.43
Counts- Tregs	115	162	238		148	184	252		0.19
Counts- γδ Tcells	56	72	105		57	83	96		0.87
Counts- NKT cells	0.17	0.33	0.65		0.15	0.31	1		0.89
Counts- iNKT cells	0.013	0.028	0.062		0.018	0.027	0.035		0.92
Counts- mDC	5.8	8.8	15		6.7	11	14		0.55

Counts- pDC	5.5	7.9	17		4.2	8.8	14		0.98
